# Aerosol Impacts on Water Relations of Camphor (*Cinnamomum camphora*)

**DOI:** 10.3389/fpls.2022.892096

**Published:** 2022-06-20

**Authors:** Chia-Ju Ellen Chi, Daniel Zinsmeister, I-Ling Lai, Shih-Chieh Chang, Yau-Lun Kuo, Jürgen Burkhardt

**Affiliations:** ^1^Institute of Crop Science and Resource Conservation, University of Bonn, Bonn, Germany; ^2^Graduate Institute of Bioresources, National Pingtung University of Science and Technology, Pingtung, Taiwan; ^3^Department of Natural Resources and Environmental Studies, Center for Interdisciplinary Research on Ecology and Sustainability, National Dong Hwa University, Hualien, Taiwan; ^4^Department of Forestry, National Pingtung University of Science and Technology, Pingtung, Taiwan

**Keywords:** stomatal conductance, vapor pressure deficit, water use efficiency, aerosol, Ball-Berry model, turgor loss point

## Abstract

Major parts of anthropogenic and natural aerosols are hygroscopic and deliquesce at high humidity, particularly when depositing to leaf surfaces close to transpiring stomata. Deliquescence and subsequent salt creep may establish thin, extraordinary pathways into the stomata, which foster stomatal uptake of nutrients and water but may also cause stomatal liquid water loss by wicking. Such additional water loss is not accompanied by a wider stomatal aperture with a larger CO_2_ influx and hypothetically reduces water use efficiency (WUE). Here, the possible direct impacts of aerosols on physical and physiological parameters of camphor (*Cinnamomum camphora*) were studied (i) in a greenhouse experiment using aerosol exclusion and (ii) in a field study in Taiwan, comparing trees at two sites with different aerosol regimes. Scanning electron microscopy (SEM) images showed that leaves grown under aerosol exclusion in filtered air (FA) were lacking the amorphous, flat areas that were abundant on leaves grown in ambient air (AA), suggesting salt crusts formed from deliquescent aerosols. Increasing vapor pressure deficit (VPD) resulted in half the Ball-Berry slope and double WUE for AA compared to FA leaves. This apparent contradiction to the wicking hypothesis may be due to the independent, overcompensating effect of stomatal closure in response to VPD, which affects AA more than FA stomata. Compared to leaves in a more polluted region in the Taiwanese Southwest, NaCl aerosols dominated the leaf surface conditions on mature camphor trees in Eastern Taiwan, while the considerably lower contact angles and the 2.5 times higher minimum epidermal conductances might have come from organic surfactants. Interpretations of SEM images from leaf surface microstructures should consider amorphous areas as possible indicators of aerosol deposition and other hygroscopic material. The amount and type of the material determine the resulting impacts on plant water relations, together with the surrounding atmosphere and ecophysiological traits.

## 1. Introduction

Atmospheric aerosols are liquid, solid, or mixed suspensions of heterogeneous chemical composition, ranging from a few nanometers to almost 100 μm in diameter (Burkhardt and Grantz, 2017). Natural atmospheric aerosols can be beneficial for plants as they carry nutrients (Chadwick et al., 1999), but in many regions aerosol concentrations are dominated by emissions from anthropogenic sources and may negatively influence both environments and organisms (Pariyar and Noga, 2018). On both global and regional scales, previous studies have long focused on the indirect impacts that atmospheric aerosols bring to plants such as the impact on water cycle, changes in radiation balance, and nutrient transport (Mahowald et al., 2017); it has been shown that the scattering of radiation caused by aerosols contributes to the photosynthesis efficiency of canopy and stem growth, and that the micro-environment near the ground also affects plant dry matter accumulation and water utilization (Liu et al., 2016; Wang et al., 2018). On the other hand, recent research has started paying more attention to the direct impact of aerosols on plants, mostly centering on the hygroscopic action of accumulated deposited aerosols on foliage. Hygroscopic particulate salts on leaf surfaces facilitate the formation of microscopic leaf wetness, may cause “wax degradation” symptoms, and affect the trace gas exchange in plants (Burkhardt and Pariyar, 2014; Coopman et al., 2021; Katata and Held, 2021); moreover, the aerosols deposited close to transpiring stomata become mobile by deliquescence and form highly concentrated solutions that may enter the stomata and connect with the liquid water that forms the end of the hydraulic system. This process (i.e., hydraulic activation of stomata, HAS) leads to liquid stomatal water loss; it is not accompanied by larger stomatal aperture and compensating CO_2_ influx, so it can be considered unproductive transpiration with a negative impact on water use efficiency (WUE; Burkhardt, 2010; Song et al., 2015; Burkhardt and Grantz, 2017). However, such an impact has not been consistently confirmed by experiment (Pariyar et al., 2013; Burkhardt and Pariyar, 2016).

Since the hygroscopic action is proposed as a primary factor of aerosol impact on plants (Burkhardt et al., 2018), and the stomata play a key role in the adaptation to changing environmental conditions (Berry et al., 2010; Bauerle and Bowden, 2011; Miner and Bauerle, 2017), this study focused on the stomatal response to the impact of aerosols, as well as its consequences for plant water relations and CO_2_ assimilation. Leaf-level physiological differences between *Cinnamomum camphora* (camphor) seedlings, grown under the exposure of aerosols and the elimination of aerosols, were compared, and similarly, the situation of mature camphor trees was studied at two Taiwanese field sites with different aerosol concentrations.

The camphor tree is a well-known versatile tree species growing in eastern Asia. The leaves are rich in bioactive compounds, and the extracted compounds are extensively used in medical treatments. With antifungal activities, the timbers of camphor are often used as building materials and furniture. Based on these characteristics and additional historical influences, camphor has become one of the most important evergreen species in Taiwan, as well as in many other tropical and subtropical areas close by Hsieh (1981); Zhou and Yan (2016); Li et al. (2020). On the other hand, the regional aerosol distribution pattern in Taiwan is strongly related to industry, geography, and season. The high density of the population and the subsequent industrial development causes higher anthropogenic aerosol emissions in western Taiwan (Tsai and Kuo, 2005; Kishcha et al., 2018). Due to the natural barrier formed by the Central Mountain Range, eastern Taiwan has relatively small air pollution. The seasonal difference in aerosol concentrations is most likely caused by the meteorological phenomena that dominate the dispersion of aerosols, and particularly the NaCl concentration varies with distance to the sea (Tsai and Chen, 2006; Chou et al., 2010; Fang and Chang, 2010). Based on the information above, the research species was chosen and the field sites in Taiwan were defined.

In this study, the aerosol loading of camphor leaves was accessed by scanning electron microscopy (SEM) and quantification of water soluble and insoluble particulate matter from leaf washing. The light saturated photosynthetic rate (A_*sat*_) and AC_*i*_ response curves were measured in order to ensure the comparable photosynthetic performance of plants from different environments. The physiological responses to aerosols were determined by foliar carbon isotope discrimination (δ^13^C) as a long term measure of WUE (Condon et al., 1992; Cabrera-Bosquet et al., 2007); the minimum leaf conductance (g_*min*_) as an indicator of uncontrollable water loss and, together with the leaf water potential at turgor loss (π_*tlp*_), as indicators of drought tolerance (Maréchaux et al., 2015; Duursma et al., 2019); and the proline accumulation as an additional indicator of osmotic adjustment to water deficit (Bates et al., 1973; Dolatabadian et al., 2008). The results of the gas exchange measurements were then introduced into the semi-empirical Ball-Berry model, which in the original form uses the relative humidity on the leaf surface and is coupled to a photosynthesis model (Farquhar et al., 1980; Ball et al., 1987). This model has been found to reflect differences in drought stress conditions between plants, and the slope factor g_1_ is inversely related to both WUE and carbon isotope composition during carbon assimilation (Knauer et al., 2017; Miner and Bauerle, 2017; Miner et al., 2017). The attraction of aerosols to water vapor might affect modeling outputs, mainly because the HAS mechanism creates a parallel transpiration pathway of liquid water, while the model relies on equivalent pathways of water vapor and CO_2_ (Aphalo and Jarvis, 1993; Monteith, 1995; Burkhardt, 2010). The original objective of this first study on aerosol- and HAS-caused effects under field conditions was the identification of physiological responses to aerosols on *C. camphora* in two field sites with different aerosol regimes, and their confirmation and explanation under greenhouse conditions with seedlings of the same species in filtered versus unfiltered air. Although the results did not follow the initial expectations, the study still found differential support for aerosol caused physiological responses under both field and greenhouse conditions.

## 2. Materials and Methods

### 2.1. Materials Preparation and Sampling Design

#### 2.1.1. Plant Material

Eight seedlings of camphor were prepared with an initial height of circa 60 cm. All the present leaves were marked non-destructively before the seedlings were assigned randomly and equally into one of two greenhouses for research. After the placement, all seedlings were irrigated regularly, pruned properly due to the spatial restriction, and fertilized every other week with a complete nutrient solution including micro-nutrients (Ferty 3; Planta Duengemittel GmbH, Hohenstauf, Germany). All measurements were obtained at 12–24 months after the seedlings were placed respectively into the greenhouses, with the plant height circa 150 cm, and only using leaves that developed inside the greenhouses.

#### 2.1.2. Greenhouse Growing Environment

The main research of this study was held in the greenhouses at the Institute of Crop Science and Resource Conservation of the University of Bonn, Germany. The two adjacent greenhouses were located on the margin of an urban area, near a multi-lane highway. One greenhouse was ventilated with ambient air (hereinafter called AA), and the other one was ventilated with filtered air (hereinafter called FA), with only about 1% of ambient aerosols remaining, representing the particles-removed environment (Grantz et al., 2018). The total aerosol concentrations were monitored by a cloud chamber condensation nuclei counter (TSI 3783; TSI, Shoreview, MN, USA). The relative humidity and temperature of the greenhouses were recorded every minute by a Tinytag data logger (TGP 4017, 1-Kanal Temperatur Datenlogger, Sensor NTC; Gemini, RS Components GmbH, Germany), showing that the environmental parameters and conditions besides the concentration of aerosols were very similar in both greenhouses (AA: 14.35 ± 6.66°C, 58.81 ± 16.58%RH, VPD: circa 0.86 kPa; FA: 13.48 ± 7.10°C, 51.79 ± 16.45%RH, VPD: circa 0.95 kPa).

#### 2.1.3. Field Sites in Taiwan

In addition to the greenhouse study, two sites with camphor tree plantations were chosen to verify and compare the results with. According to previous long-term monitoring results (between 2008 and 2016), the southwestern region is likely to have a higher PM_2.5_ concentration (49.14 ± 15.95 μg/m^3^) than the eastern region (15.62 ± 8.73 μg/m^3^), especially during winter time (Chen et al., 2018, 2020; Ho et al., 2020; Wang et al., 2021). Therefore, the two plantations which are located in Pingtung county (southwestern Taiwan) and Hualien county (eastern Taiwan) were chosen for the field research. Both sites are afforestation after the abandonment of a long history of sugarcane plantation and are composed of circa 15 endemic broad-leaf tree species. The 675 ha Pingtung site was planted since 2006, while the 1,250 ha Hualien site was planted since 2002. On both sites the plantations are managed and owned by the Taiwan Sugar Corporation. In order to understand the growth status of plants and their contribution to carbon sequestration, flux towers were built and research instruments were installed for monitoring (Wu et al., 2015; Maneke-Fiegenbaum et al., 2018). The canopies of camphor trees were accessed by the existing scaffoldings. In Pingtung site 3 camphor trees were accessible (7 December to 13 December 2019), and in Hualien site 4 camphor trees were accessible (28 November to 4 December 2019).

#### 2.1.4. Sampling Design and Data Analysis

An overview of measured parameters is given in Table 1. The investigations tackled physical and physiological processes, which affected statistical procedures. Measurements of physical parameters (aerosol loading, contact angle, SEM) were evaluated as single leaf data in each treatment; while measurements of physiological parameters (A_*sat*_, AC_*i*_ fitting data, δ^13^C, g_*min*_, π_*tlp*_, water potential, proline concentration, g_*sw*_ to VPD, Ball-Berry model) were evaluated with the mean value of each individual tree, then further compared between treatments. Statistical analysis was performed using R Studio (R version 4.0.3). Shapiro-Wilk test was used as a normality test for distributed data, and *F*-test was performed for comparing two variances. For normally distributed data, the significance of differences between different groups was estimated by using the Student's *t*-test. For data with non-normal distribution, statistical analysis was performed with the non-parametric method using Wilcoxon-Mann–Whitney *U*-test to find out the differences between groups. In all statistical analyses, the differences were considered significant if the *p* < 0.05.

**Table 1:** An overview of *Cinnamomum camphora* measurements in the greenhouses and the fields.

	**Greenhouse**		**Field**	
**Measurement**	**Ambient air (AA)**	**Filtered air (FA)**	**Pingtung**	**Hualien**
A_*sat*_	v	v	v	v
AC_*i*_ fitting parameters	v	v	v	v
SEM	v	v		
Particulate matter	v	v		
Dissolvable aerosols	v	v	v	v
g_*min*_	v	v	v	v
δ^13^C	v	v	v	v
π_*tlp*_	v	v	
Water potential (predawn, noon)			v	v
g_*sw*_ to VPD curves	v	v		
Contact angle	v	v	v	v
Proline content	v	v		

*The measurements include light saturated net photosynthetic rate (A_sat_), photosynthetic parameters fitted from AC_i_ response curve (the response of net CO_2_ assimilation to the CO_2_ concentration in the intercellular airspaces of the leaf), scanning electron microscopy images (SEM), aerosol loading evaluation (the concentration of not dissolvable particulate matter and dissolvable aerosols), minimum leaf conductance (g_min_), carbon isotope composition (δ^13^C), water potential at predawn, noon, turgor loss (π_tlp_), the response curve of stomatal conductance (g_sw_) to vapor pressure deficit at leaf temperature (VPD), contact angle, and proline content*.

### 2.2. Methodologies and Experimental Design

#### 2.2.1. Scanning Electron Microscopy

The amount and behavior of deposited aerosols on leaf surfaces were visualized by scanning electron microscopy (SEM, Leo 1450 VP, Zeiss, Jena, Germany), in the Nees Institute for Biodiversity of Plants of the University of Bonn, Germany (conducted in May 2021). Fresh leaves were obtained from camphor seedlings grown in AA and FA greenhouses and transferred immediately into the laboratory. All samples were taken from fully expanded and dark-green leaves from the 120 cm height of the plants. Due to the requirement of a conductive coating on the surface of samples, the samples of both adaxial and abaxial sides were covered by a commonly-used palladium coating for high-vacuum SEM imaging (Achneck et al., 2010). While interpreting the SEM images, the edges of the pictures were avoided due to the possible instabilities caused by the limitation and disturbance from the instrument.

#### 2.2.2. Aerosol Loading

The concentration of deposited aerosols on leaf surfaces was determined by foliar rinsing. Each leaf sample was taken pictures before placing into falcon tubes with 40 ml of Millipore water. Without the petiole steeped in the deionized water, falcon tubes were brought to ultrasonic baths (SONOREX, BANDELIN electronic GmbH & Co. KG, Berlin, Germany) for 5 min at 30°C. After taking out the washed leaves, the solution in each falcon tube was filtered with a pore size 0.45 μm and outer diameter 33 mm syringe filter (Carl Roth GmbH & Co. KG, Karlsruhe, Germany) in order to remove the not dissolvable particulate matter (Dzierżanowski et al., 2011; Chen et al., 2022). The filter was weighed before and after filtering to measure the amount of not dissolvable particulate matter deposited on leaf surfaces. The ion concentrations of the solution in falcon tubes were then measured using ion chromatography (Cl^−^, ${\text{NO}}_{3}^{-}$, ${\text{SO}}_{4}^{2-}$), atomic absorption spectrometer (Mg^2+^), flame photometer (Na^+^, K^+^, Ca^2+^), and a continuous flow analyzer with photometric detection (${\text{NH}}_{4}^{+}$) (Burkhardt and Pariyar, 2016). For calculating the ion concentration based on the unit of certain leaf area (including both adaxial and abaxial sides of leaf surface), ImageJ was used to analyze the leaf area of samples (Schneider et al., 2012; Grantz et al., 2018). The measurements of the greenhouse study were conducted in March 2021 (AA: *n* = 12, FA: *n* = 9); field research was in November to December 2019 (Pingtung: *n* = 17, Hualien: *n* = 12).

#### 2.2.3. Contact Angle

In the greenhouse study, contact angles of 1-μl droplets of water on the cuticles were measured by a goniometer (DSA 30E; Kruess GmbH, Hamburg, Germany). Fully expanded fresh leaves were harvested from a 120 cm height of camphor seedlings in both greenhouse AA and FA (AA: *n* = 12, FA: *n* = 9; conducted in February 2021). The surface tension of the solution was determined by the pendant drop method and shown as angles (Burkhardt et al., 2012). In the field research in December 2018 (Pingtung: *n* = 8, Hualien: *n* = 16), the droplets of water were manually applied on the leaf surfaces and the images were captured by a portable microscope (DigiMicro Profi, dnt Innovation GmbH, Germany). The contact angles were then calculated with ImageJ (Schneider et al., 2012).

#### 2.2.4. Photosynthetic Parameters

Photosynthetic light response curve and AC_*i*_ response curve [the response of net CO_2_ assimilation (A) to the CO_2_ concentration in the intercellular airspaces of the leaf (C_*i*_)] were measured by LI-6400 and LI-6800 Portable Photosynthesis System (LI-COR Biosciences, Lincoln, NE, USA) on fully expanded leaves at 120 cm height. For the light response curve, measurements began with the saturating irradiance (1,400 μmol m^−2^ s^−1^) followed by the reductions of 1,400, 550, 200, 100, 50, 20 μmol m^−2^ s^−1^, until the irradiance was 0 μmol m^−2^ s^−1^. The other environmental settings remained as leaf temperature close to environment temperature, leaf vapor pressure deficit (VPD_*leaf*_) circa 1.5–2 kPa, and chamber CO_2_ concentration 400 μmol mol^−1^. Light saturated net photosynthetic rate (A_*sat*_) was then defined as the net CO_2_ assimilation (A) at irradiance 1,400 μmol m^−2^ s^−1^ (Herrick and Thomas, 1999; Oliveira and Peñuelas, 2005; Sazeides et al., 2021). On the other hand, before measuring the AC_*i*_ response curve, leaves were acclimated to saturating irradiance (1,400 μmol m^−2^ s^−1^) for 30 min with leaf temperature 20°C, VPD_*leaf*_ 1.5 kPa, and flow rate 300 μmol s^−1^. Without changing the above environmental settings, net CO_2_ assimilation rate (A) was measured at a sequence of chamber CO_2_ concentrations: 400, 300, 200, 100, 50, 400, 400, 400, 600, 800, 1,000, 1,200, 1,600, 2,000 μmol mol^−1^ (Feng and Dietze, 2013). Afterward, maximum carboxylation rate of Rubisco (V_*cmax*_), maximum rate of electron transport for the given light intensity (J), maximum rate of triose phosphate use (TPU), daytime respiration (R_*d*_), and mesophyll conductance to CO_2_ transfer (g_*m*_) were fitted with an Excel spreadsheet tool published in previous research (Sharkey et al., 2007). The measurements of the greenhouse study were conducted in May 2021 (*n* = 4); field research was in November to December 2019 (Pingtung: *n* = 3, Hualien: *n* = 4).

#### 2.2.5. Carbon Dioxide Discrimination

The carbon isotope composition was measured with an isotope ratio mass spectrometer (IRMS, C-N-S Analyzer, and MS-2020; SerCon Ltd., Crewe, UK). Three leaves from 120 cm height of camphor seedlings were taken for each sample. The harvested leaves were dried in a laboratorial oven at 60°C for 1 week to reach the absolute dry weight and were ground to a fine powder. 1±0.1 mg of ground samples were weighed with an electronic micro-balance (M2P, Sartorius Lab Instruments GmbH & Co. KG, Goettingen, Germany) and loaded into tin capsules. During operation in the C-N-S Analyzer, the tin capsule reached 1,800°C and fell into the combustion furnace as CO_2_ was injected. Soon after oxidation, the sample went through a purification process (Cr_2_O_3_, CuO, Ag-wool layer) with He carrier gas, in order to assure the complete oxidation and removal of unnecessary S in the sample. The sample then passed through the reduction furnace containing Cu at 600°C, where the excess CO_2_ and H_2_O were removed. The resulting gas stream was carried to a gas chromatography column and then the separated CO_2_ was brought to the mass spectrometer. During operation in the mass spectrometer, the inlet gas stream was ionized as an ion beam and was separated by a permanent magnet while passing through the passage, and then reached the final isotope detector. From the ratio of signals which were collected at the detector, the ^13^C value was calculated. The carbon isotope composition (δ^13^C) was then calculated by comparison to a standard (Condon et al., 2002; Burkhardt and Pariyar, 2016). The measurements of the greenhouse study were conducted in March 2021 (*n* = 3); field research was in November to December 2019 (Pingtung: *n* = 3, Hualien: *n* = 4).

#### 2.2.6. Minimum Leaf Conductance

The samples of camphor were taken from fresh leaves and then immediately brought to the lab (*n* = 4; conducted in March 2021). After sealing the basis of the petiole to prevent the water loss from the petiole, the samples were labeled and the pictures of leaf surfaces were taken for calculating the leaf area with a known scale by ImageJ (Schneider et al., 2012). During dehydration, the samples were hung on a framework with proper spaces separating the leaves in a ventilated fume hood. The samples were weighed on a digital semi-micro balance (EX125M, EXPLORER^Ⓡ^ SEMI-MICRO, Ohaus Corporation, Parsippany, NJ, USA) once an hour, meanwhile both the temperature and humidity of the drying environment were continuously recorded by a Tinytag data logger. This process was repeated for about 72 h, with 6–8 measurements in the linear part of the regression line. The modified Arden Buck equation (Buck, 1981, 1996) was used to calculate the saturated vapor pressure (VP_*sat*_, kPa). Together with the leaf drying weight, relative humidity, temperature, and leaf area, g_*min*_ values were finally calculated by the spreadsheet tool (Sack and Scoffoni, 2011). The mean g_*min*_ value of each sample was calculated by the 6–8 measurements from the linear part of the regression line in the graph, which was supposed to be close to the g_*min*_ value calculated by the slope in the graph. In order to compare the differences between different groups, the g_*min*_ values were then statistically analyzed.

#### 2.2.7. Leaf Water Potential

The leaf water potential at turgor loss (π_*tlp*_) is strongly related to plant drought tolerance (Maréchaux et al., 2015). Instead of the standard pressure–volume (p–v) curve approach, using an osmometer is one of the most rapid and reliable methods to predict π_*tlp*_ (Bartlett et al., 2012a). In the greenhouse study (AA: *n* = 4, FA: *n* = 3; conducted in March 2021), branches from a certain height of the plants were cut and quickly placed into water, and then cut again underwater at least 2 cm distal to the original cut. This standard pre-treatment of rehydration was covered by a black plastic bag and performed overnight (from sunset to shortly after sunrise) 1 day before measuring. The next morning, the branches were wrapped slightly in a wet paper towel and placed in zipper bags while transferring to the lab. The bags were then stored in the fridge, with only one leaf sample taken out each time for measurements. One leaf disc was taken from one mature and fully expanded leaf per branch. The discs were taken in the middle between the midrib and margin and between the leaf tip and base, using a 6 mm diameter cork borer and avoiding secondary veins. The leaf disc was then immediately folded inside the foil square (3 × 3 cm^2^) and frozen in liquid nitrogen for 2 min in order to fracture the cell walls. Afterward, the leaf disc was punctured using tweezers 10–12 times and then rapidly sealed in the vapor pressure osmometer (VAPRO 5600, Wescor, Inc, Logan, UT, USA). The osmolality (mmol kg^−1^) was measured after the values reached equilibrium (8–12 min waiting time). The osmotic potential (π_*o*_) was then calculated by using osmolality obtained from the vapor pressure osmometer of freeze-thawed leaf discs, following Van't Hoff Equation (1) which relates solute concentration to vapor pressure:


(1) $$\begin{array}{r} \pi_{o}=-C_{o}\times R\times T \end{array}$$

where C_*o*_ is the molar solute concentration (mmol kg^−1^), R is the universal gas constant 8.3144598E-0.6 (m^3^ MPa K^−1^ mol^−1^), T is the temperature (K) (Khare, 2015). Due to the strong correlation between π_*o*_ and π_*tlp*_ (Bartlett et al., 2012a), π_*tlp*_ was then calculated from π_*o*_ by using the adapted regression Equation (2) from previous research (Bartlett et al., 2012b; Sjöman et al., 2015; Banks and Hirons, 2019):


(2) $$\begin{array}{r} \pi_{tlp}=-0.2554+1.1243\times \pi_{o} \end{array}$$

where the *R*^2^ of this π_*tlp*_ prediction from π_*o*_ is proposed as 0.91.

In the field study, the leaf water potential was measured at predawn and noon time in the Pingtung site and Hualien site (*n* = 3 and 4, respectively). A small twig with leaves was cut off from individual camphor trees with aluminum foil slightly wrapped in order to prevent water loss; the twig was then immediately transferred into Scholander Pressure Chamber (Model 3005, Soil Moisture Equipment Corp., Santa Barbara, CA, USA) for measuring (Pariyar et al., 2013; Kuo et al., 2017).

#### 2.2.8. Proline Concentration

Fully expanded fresh leaves were harvested from a 120 cm height of camphor seedlings in both greenhouse AA and FA (AA: *n* = 4, FA: *n* = 3; conducted in April 2021). Five samples were taken from each seedling, and each sample contained 1–2 leaves depending on the leaf size. Samples were placed separately in zipper bags at −20°C for deep-freezing. Afterward, samples were freeze-dried for 2 days under vacuum without thawing (ALPHA 1-4 LDplus/ALPHA 2-4 LDplus, Martin Christ Gefriertrocknungsanlagen GmbH, Osterode am Harz, Germany). The samples were then ground into a fine powder (Mixer Mill MM 301, Retsch GmbH, Haan, Germany) and weighed 100 mg per sample with an analytical balance (BP 210 S, Sartorius Lab Instruments GmbH & Co. KG, Goettingen, Germany).

For the extraction, 3 ml of 3% sulfosalicylic acid was added to each sample. The samples were then shaken for 20 s and centrifuged at room temperature for 20 min at 4,200 rpm. For each sample, 2 ml of supernatant solution, 2 ml of glacial acetic acid (100%), and 2 ml of ninhydrin acid solution (ninhydrin mixed with glacial acetic acid and orthophosphoric acid) were mixed in a clean test tube. After being shaken homogeneously, the samples were placed in a hot-bath (100°C) for 1 h to boost the chemical reaction and then brought into an ice-bath to stop the chemical reaction until they reached room temperature. Four milliliters of toluene was added to each test tube, and the test tube was closed tightly with a rubber plug before mixed on a vortex mixer for 30 s. In order to get stratification, the test tube was left standing for 15 min until the toluene and aqueous phases were separated distinctly. The toluene phase (red-colored, upper part) was then carefully transferred into a half micro-acryl cuvette, and the absorbance of the solution was measured with a spectrophotometer at wavelength 520 nm (Lambda 35 UV/Vis Spectrophotometer, Perkin Elmer LAS GmbH, Solingen, Germany). The concentration of proline was calculated from a proline standard curve following Equation (3) and was expressed as μeq g^−1^ dry matter (Dolatabadian et al., 2008; Pariyar and Noga, 2018).


(3) $$\begin{array}{r} Proline\;\left(\mu molg^{-1}drymatter\right)=\frac{\left(A_{520nm}-b\right)/a\times V\times DF}{M_{proline}\times Wt_{sample}} \end{array}$$

In Equation (3), A_520*nm*_ is the absorbance of the solution at wavelength 520 nm, a and b are the coefficients of slope and intercept from the linear equation (*y* = *ax*+*b*) of the standard proline concentrations gradient curve, V is the volume of sulfosalicylic acid (3 ml), DF is the dilution factor (1.5), the ratio of sulfosalicylic acid and supernatant solution, M_*proline*_ is the molecular weight of proline (115.5 g mol^−1^), and Wt is the weight of the initial sample (0.1 g).

#### 2.2.9. Stomatal Conductance to Water Vapor

The gas exchange measurements were conducted in the greenhouses during cloudy days in winter (February 2021) in order to reduce the influence of circadian changes (Grantz et al., 2018). The response curve of stomatal conductance (g_*sw*_) to vapor pressure deficit at leaf temperature (VPD) was determined using a steady-state gas exchange system (LI-6800). The photosynthetic photon flux density (PPFD) incident on the leaf (i.e., Q_*in*_) was set as 500 μmol m^−2^ s^−1^ to avoid over saturation. Sample cell CO_2_ concentration was set as 400 μmol mol^−1^, flow rate to the chamber as 300 μmol s^−1^, chamber fan rotation rate as 14,500 rpm, and leaf temperature as 15°C (evaluated by the ambient environment and temperature restriction). Considering the sensibility of g_*sw*_ to changing VPD and the instrument limitation of CO_2_ supply, the sample was measured at a stepwise sequence of VPD: 0.50, 0.75, 1.00, 1.25, 1.50 kPa. Before switching to the next VPD set point, the gas analyzers of the sample and reference were matched to assure accuracy and stability. With each VPD, measurements were recorded every minute until the photosynthetic parameters reached equilibrium, resulting in a 40-min to 2-h acclimation. For data collection, the mean of the last 10 measurements of each VPD was taken for further statistical analysis. Not only the response curve of stomatal conductance to increasing vapor pressure deficit was displayed, but also the parameter g_*sw*_ was performed according to the Ball-Berry model (Equation 4). This model presents g_*sw*_ as a function of assimilation (A_*n*_), relative humidity (H_*s*_), and CO_2_ concentration at the leaf surface (C_*s*_).


(4) $$\begin{array}{r} g_{sw}=g_{1}\times A_{n}\times \frac{H_{s}}{C_{s}}+g_{0} \end{array}$$

The g_*sw*_ results from the linear approach, where the slope constant (g_1_) is the slope of the relationship between g_*sw*_ and ${\text{A}}_{n}{\;}^{*}\;$H_*s*_/C_*s*_ (i.e., Ball Index), and g_0_ comes from the intercept when A_*n*_ is zero. The slope represents a compromise between the costs and benefits of g_*sw*_ relative to the photosynthetic activity of the leaf (Ball et al., 1987; Medlyn et al., 2017; Miner and Bauerle, 2017).

## 3. Results

### 3.1. SEM Images

**Figure 1:**
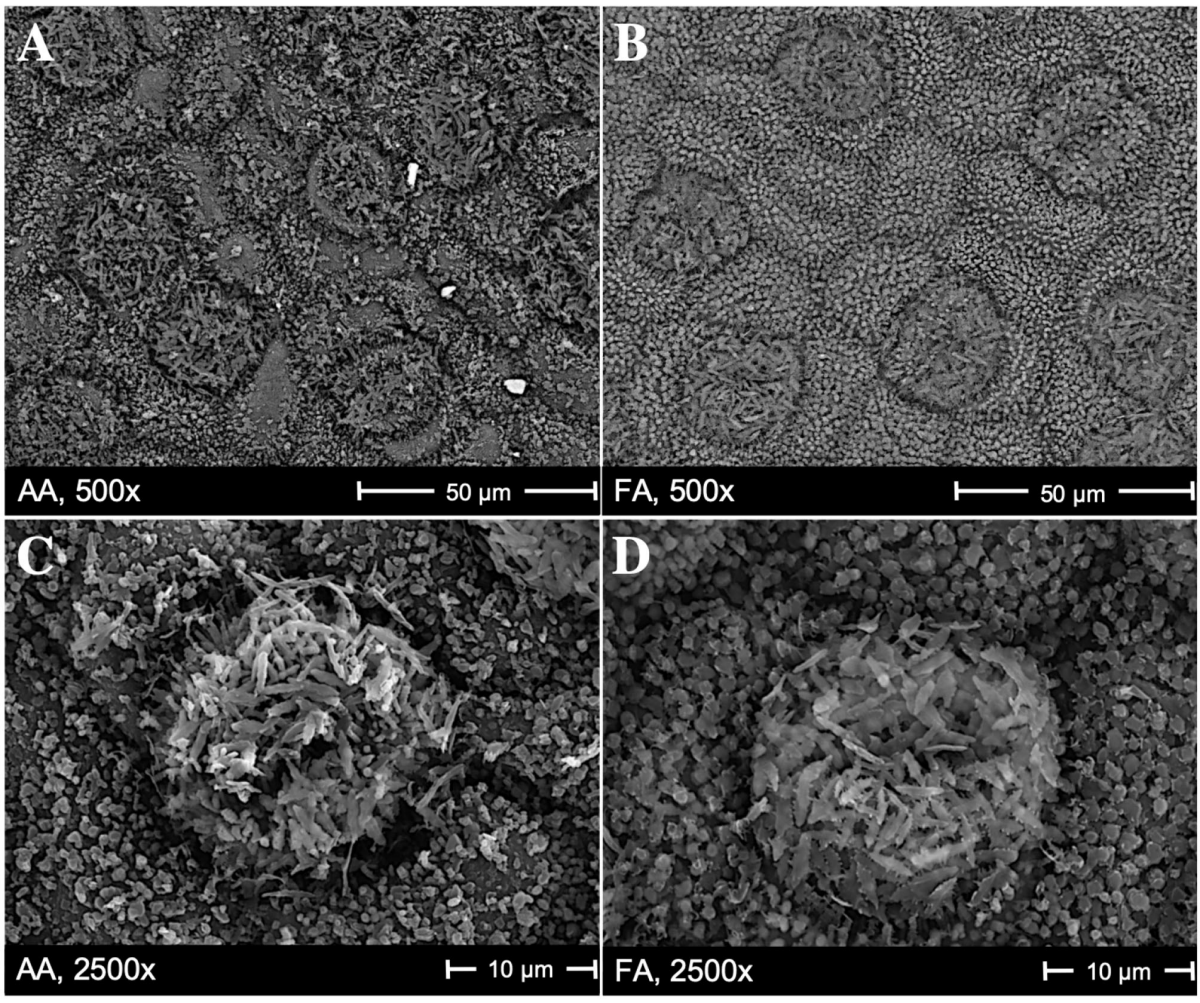
Scanning electron microscopy images showing the stomata patterns on the abaxial surface of *Cinnamomum camphora*, with magnification 500x and 2,500x. **(A,C)** are from AA leaves (the greenhouse with ambient air) and **(B,D)** are from FA leaves (the greenhouse with filtered air). Flat, amorphous areas in **(A,C)** are probably caused by deliquescent, hygroscopic aerosols. Such an area is, e.g., in **(A)** above the left part of the 50 μm scale.

Scanning electron microscopy images in Figure 1 show the cuticular and stomatal patterns on abaxial surfaces of *C. camphora* leaves, with clear differences in the microstructures of AA leaves (Figures 1A,C) compared to FA leaves (Figures 1B,D). On the surfaces of AA leaves, there are more particles deposited visibly, which are shown as non-transparent, brighter, and randomly distributed granules in the SEM images, compared to FA. Flat, amorphous areas are only observed on AA leaf surfaces (Figures 1A,C), and may indicate salt crusts resulting from hygroscopic aerosols after deliquescence. Around these flat areas, the wax crystals are faintly covered; additionally, the original wax structures of stomata and epidermal cells are changed in AA leaves. In Figure 1C, it is visible that the arrangement of wax on the stomata and surrounding cells is less neatly distributed than in Figure 1D. Their appearance supports the hypothesis of the hygroscopic layer formed by deliquescent aerosols, which resulted in the overall impression of more disturbed surfaces, less defined stomatal structures, and a less visible stomata distribution for AA compared to FA leaves.

### 3.2. Deposited Aerosol Concentration on Leaf Surfaces

**Figure 2:**
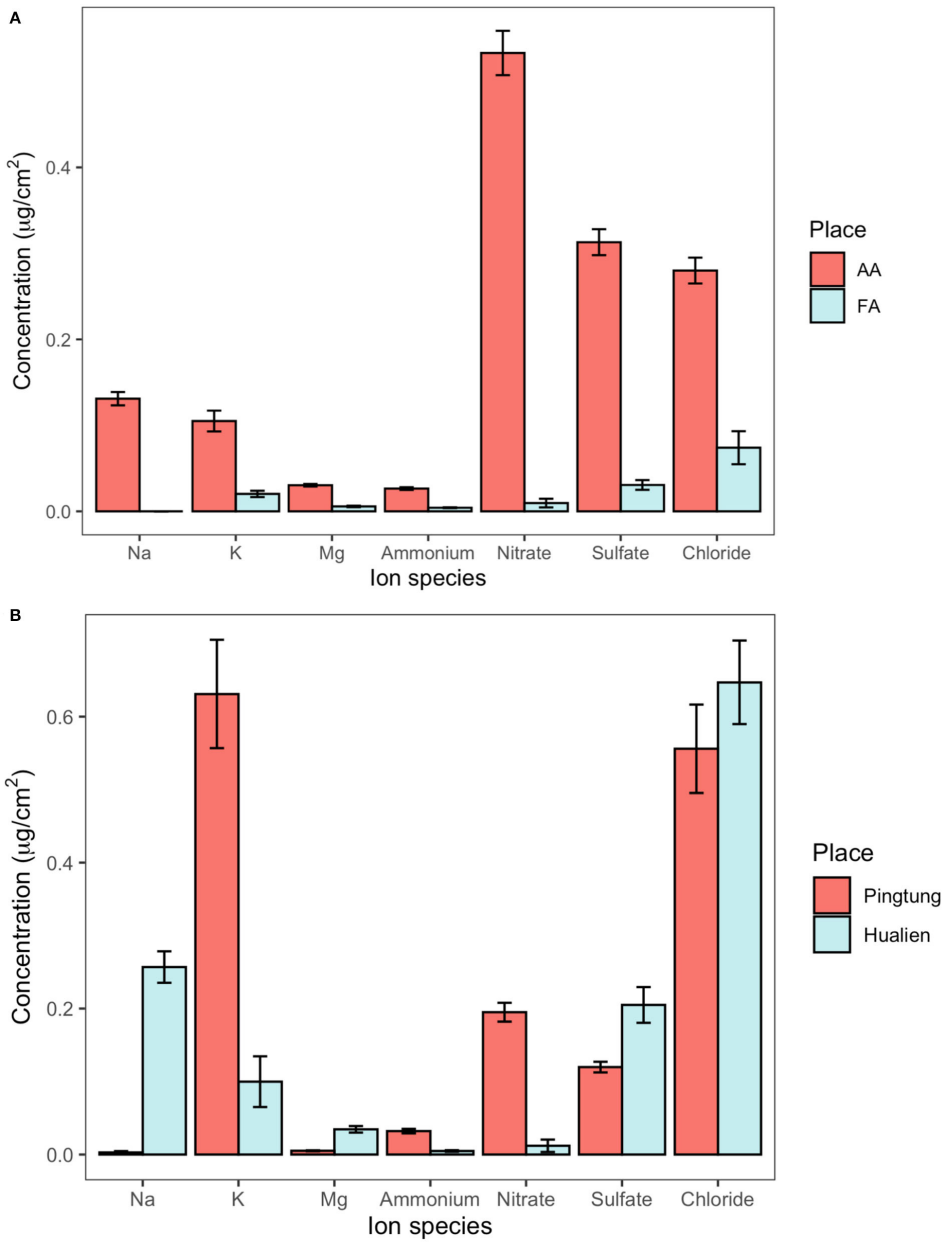
The concentration of dissolvable aerosols deposited on leaf surfaces from greenhouses and fields, determined by foliar rinsing. **(A)** Ion concentration on camphor leaves from the AA (unfiltered, ambient air) and FA (filtered air) greenhouses. **(B)** Ion concentration on camphor leaves at Pingtung and Hualien field site.

Table 2 shows the concentration of not dissolvable particulate matter and the overall amount of dissolvable aerosols deposited on leaf surfaces in greenhouse AA and FA, each number referring to the total adaxial and abaxial leaf area. The weight of not dissolvable particulate matter deposited on AA leaves was higher than on FA leaves, with the comparable median value of 3.59 μg/cm^2^ (*n* = 12) and 1.40 μg/cm^2^ (*n* = 9), respectively. The total amount of dissolvable aerosols in AA was more than 9 times higher than in FA. The ratio of not dissolvable particulate matter to total deposited aerosol amount within a square centimeter in AA is 74%, and in FA is 93%. Figure 2A details the ionic composition of the dissolvable aerosols, respectively. Nitrate, sulfate, and chloride are the dominant compounds of aerosol deposition in AA, while Na, K, Mg, and ammonium are the subordinate ones. In FA, the concentration of chloride, sulfate, and K is relatively higher than the other ions. Figure 2B shows the concentration of dissolvable aerosols deposited on leaf surfaces from field sites in Taiwan. The dominant compounds in Pingtung are K and Cl, which are much higher than the concentration of nitrate, sulfate, and ammonium. Mg and Na show a value close to zero of the concentration in Pingtung. A different distribution pattern is found in Hualien, with Cl having the highest concentration, followed by Na, sulfate, K, Mg, nitrate, and ammonium. Although there are differences between compound species in Pingtung and Hualien, the total concentration of dissolvable deposited aerosols in Pingtung (1.54 ± 0.142 μg/cm^2^, *n* = 17) is not significantly higher than in Hualien (1.26 ± 0.132 μg/cm^2^, *n* = 12).

**Table 2:** Measurements of *Cinnamomum camphora* leaves from different growing environments.

		**Greenhouse**			**Field**		

**Measurement**	
		**AA**	**FA**	**Significance**	**Pingtung**	**Hualien**	**Significance**
A_*sat*_	(μmol m^−2^ s^−1^)	6.70 ± 0.90	7.29 ± 1.37	*p* = 0.731	12.30 ± 3.27	15.30 ± 1.12	*p* = 0.367
V_*cmax*_	(μmol m^−2^ s^−1^)	102.11 ± 9.05	111.21 ± 8.02	*p* = 0.480	102.42 ± 17.97	74.53 ± 9.02	*p* = 0.191
J	(μmol m^−2^ s^−1^)	135.89 ± 7.08	138.83 ± 8.06	*p* = 0.793	113.06 ± 12.28	99.52 ± 7.45	*p* = 0.363
TPU	(μmol m^−2^ s^−1^)	10.69 ± 0.41	10.73 ± 0.59	*p* = 0.961	8.32 ± 0.82	7.82 ± 0.71	*p* = 0.667
R_*d*_	(μmol m^−2^ s^−1^)	8.73 ± 1.16	8.00 ± 1.82	*p* = 0.747	0.93 ± 0.15	0.85 ± 0.07	*p* = 0.583
g_*m*_	(μmol m^−2^ s^−1^ Pa^−1^)	14.24 ± 4.19	5.03 ± 2.23	*p* = 0.100	19.73 ± 9.44	22.00 ± 5.31	*p* = 0.831
Particulate matter	(μg/cm^2^)	3.59	1.40	*p* <0.01
Dissolvable aerosols	(μg/cm^2^)	1.42 ± 0.06	0.15 ± 0.03	*p* <0.001	1.54 ± 0.14	1.26 ± 0.13	*p* = 0.177
g_*min*_	(mmol m^−2^ s^−1^)	0.49 ± 0.03	0.48 ± 0.03	*p* = 0.967	0.99 ± 0.13	2.46 ± 0.20	*p* <0.01
δ^13^C		−28.10 ± 0.20	−27.70 ± 0.55	*p* = 0.510	-31.58 ± 0.50	-32.99 ± 0.40	*p* = 0.077
π_*tlp*_	(MPa)	−3.43 ± 0.09	−3.26 ± 0.08	*p* = 0.210	
Water potential, predawn	(MPa)				−0.09 ± 0.01	−0.10 ± 0.02	*p* = 0.840
Water potential, noon	(MPa)				−0.64 ± 0.10	−0.52 ± 0.03	*p* = 0.210
Contact angle, adaxial	(°)	124.56 ± 2.62	127.00 ± 2.47	*p* = 0.520	65.96 ± 7.59	53.16 ± 1.87	*p* = 0.093
Contact angle, abaxial	(°)	140.13 ± 1.37	143.14 ± 1.18	*p* = 0.128	119.33 ± 9.47	75.68 ± 5.13	*p* <0.001
Proline content	(μmol g^−1^)	1.57 ± 0.57	1.22 ± 0.39	*p* = 0.659	

*AA is the greenhouse with ambient air and FA is the greenhouse with filtered air; Pingtung is the expectedly more polluted field and Hualien is the expectedly less polluted field. The results show the key photosynthetic parameters (light saturated net photosynthetic rate (A_sat_), maximum carboxylation rate of Rubisco (V_cmax_), maximum rate of electron transport for the given light intensity (J), maximum rate of triose phosphate use (TPU), daytime respiration (R_d_), and mesophyll conductance to CO_2_ transfer [g_m_)], the concentration of not dissolvable particulate matter, total concentration of dissolvable deposited aerosols, minimum epidermal conductance (g_min_), carbon isotope composition (δ^13^C) values, leaf water potential at turgor loss (π_tlp_), predawn, noon, contact angles, and proline concentration. The values are presented as mean±SE (statistically analyzed with Student's t-test), besides the values of particulate, which are presented as median (statistically analyzed with Wilcoxon-Mann–Whitney U-test). Sample size and research conducted time for each measurement are indicated in the text. Statistical significance is shown with the p-value*.

### 3.3. Contact Angle

Table 2 shows the difference in contact angles on adaxial and abaxial leaves from greenhouse AA and FA, as well as of leaves from the fields. There is no significant difference in adaxial contact angles between AA (124.56 ± 2.62, *n* = 12) and FA (127.00 ± 2.47, *n* = 9) leaves, neither between Pingtung (65.96 ± 7.59, *n* = 14) and Hualien (53.16 ± 1.87, *n* = 16). With abaxial contact angles, AA leaves (140.13 ± 1.37, *n* = 12) and FA leaves (143.14 ± 1.18, *n* = 9) do not differ either. However, Pingtung leaves (119.33 ± 9.47, *n* = 8) have higher values than Hualien leaves (75.68 ± 5.13, *n* = 16).

### 3.4. Photosynthetic Parameters

In Table 2, the key photosynthetic parameters of leaves from AA and FA are presented (*n* = 4). There are no significant differences in A_*sat*_, V_*cmax*_, J, TPU, R_*d*_, and g_*m*_ between the leaves from two greenhouses, nor between the two field sites.

### 3.5. Plant Water Relations and Drought Tolerance Measurements

#### 3.5.1. Carbon Dioxide Discrimination

In Table 2, δ^13^C values are generally less negative in the greenhouses than in the fields, but the results between more polluted and less polluted environments are not consistent. It is noted that there is a tendency toward lower values at Hualien compared to Pingtung, although the comparison is not useful (refer to below). Between the different greenhouses, where the isotope ratio could possibly allow comparison of long-term stomatal aperture due to equal environmental conditions, there is no significant difference in δ^13^C between AA (−28.10 ± 0.20) and FA (−27.70 ± 0.55), respectively (*n* = 3).

#### 3.5.2. Minimum Leaf Conductance

There is no significant difference in g_*min*_ of *C. camphora* leaves between AA (*n* = 4) and FA (*n* = 4). The g_*min*_ value of leaves in AA shows 0.49 ± 0.03 mmol m^−2^ s^−1^, very close to the g_*min*_ value of leaves in FA which is 0.48 ± 0.03 mmol m^−2^ s^−1^. On the other hand, the g_*min*_ of leaves from Pingtung (*n* = 3) is found much lower than in Hualien (*n* = 4), with the value of 0.99 ± 0.13 mmol m^−2^ s^−1^ and 2.46 ± 0.20 mmol m^−2^ s^−1^, respectively (Table 2).

#### 3.5.3. Leaf Water Potential at Predawn, Noon, and Turgor Loss

There is no significant difference in leaf water potential at turgor loss (π_*tlp*_) in the greenhouses. Predawn and noon leaf water potential at the field sites are not significantly different (Table 2), supporting comparable water status during the measurement campaign.

#### 3.5.4. Proline Concentration

There is no significant difference in proline concentration of *C. camphora* leaves between AA (*n* = 4) and FA (*n* = 3). The accumulated proline content of leaves in AA is 1.57 ± 0.57 μmol g^−1^, and in FA it is 1.22 ± 0.39 μmol g^−1^ (Table 2).

### 3.6. Stomatal Conductance to Water Vapor

**Figure 3:**
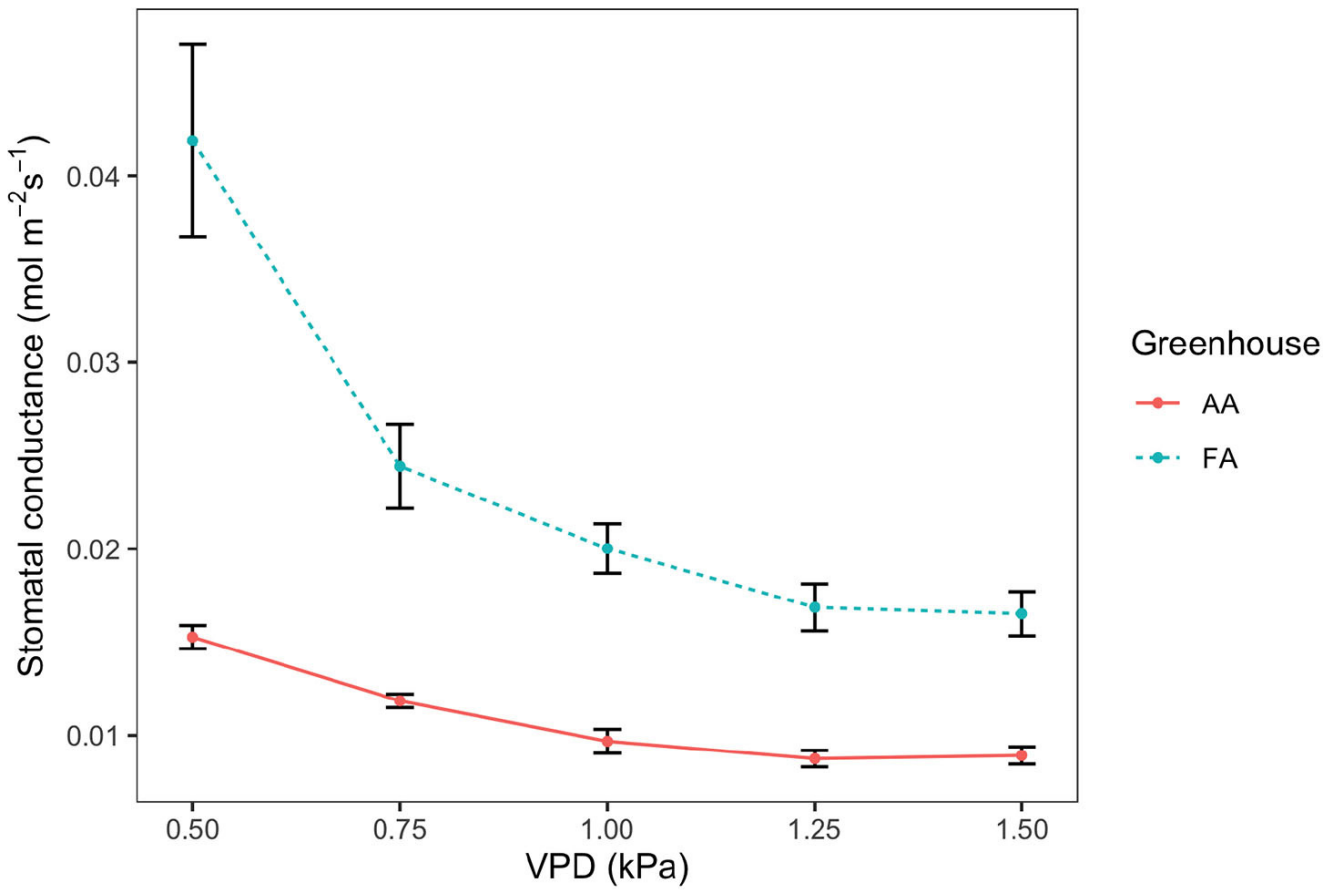
Stomatal conductance (g_*sw*_) to vapor pressure deficit (VPD) response curve, for leaves from greenhouse AA (ambient air) and greenhouse FA (filtered air). The points and error bars represent mean ± SE (*n* = 3). The solid line is AA and the dashed line is FA.

**Figure 4:**
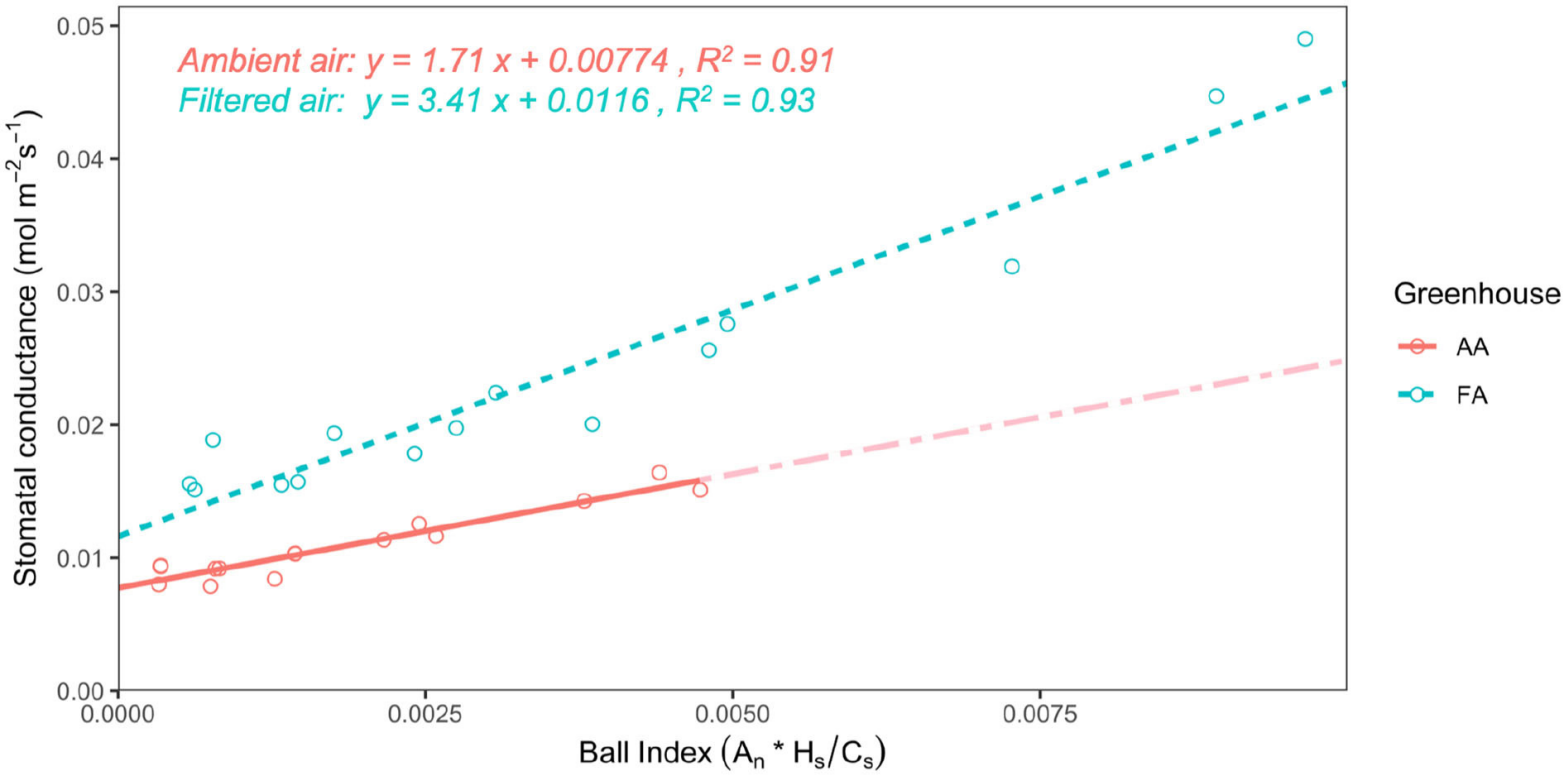
Relationship of stomatal conductance with the Ball Index for leaves from greenhouse AA (ambient air) and greenhouse FA (filtered air). The linear regressions of the Ball-Berry model represent the means of linear functions fitted to data from individual leaves at all measured vapor pressure deficit (VPD) levels; the Ball Index is calculated with assimilation (A_*n*_), relative humidity (H_*s*_), and CO_2_ concentration at the leaf surface (C_*s*_). The solid line is AA, with a partially dash-dotted line showing the extension to the full range of the x-axis, based on predicted data points from the linear regression; and the dashed line is FA. A statistical analysis of the slope and intercept indicates a significant difference in the Ball-Berry model between leaves from AA and FA (*P* < 0.005).

Stomatal conductance (g_*sw*_) shows a decreasing tendency as VPD increases, both in AA and FA (Figure 3; *n* = 3, respectively). In AA, the g_*sw*_ value decreases more moderately from 0.015 ± 0.001 mol m^−2^ s^−1^ (while VPD 0.50 kPa) to 0.009 ± 0.0005 mol m^−2^ s^−1^ (while VPD 1.50 kPa), with small SEs. However, in FA, the g_*sw*_ value falls more rapidly from 0.042 ± 0.005 mol m^−2^ s^−1^ (while VPD 0.50 kPa) to 0.017 ± 0.001 mol m^−2^ s^−1^ (while VPD 1.50 kPa). With each VPD set point, leaves in AA show a lower g_*sw*_ value than in FA (*p* < 0.01), especially when VPD is low (i.e., 0.50 kPa). Additionally, it is shown that in both AA and FA, the g_*sw*_ of leaves maintain a similar value instead of decreasing while VPD changes from 1.25 to 1.50 kPa. Subsequently, differences are also found for the stomatal model parameters g_0_ and g_1_, calculated from the Ball Index on the basis of assimilation (A_*n*_), relative humidity (H_*s*_), and CO_2_ concentration at the leaf surface (C_*s*_), and then further performed with the Ball-Berry model (Figure 4). The values of A_*n*_ in AA are generally lower than FA, causing a lower range of the Ball Index. The regression line of AA leaves is, therefore, extended to the full range of the x-axis by using the data points predicted with the linear model; and two regression lines are compared based on the actual data points. Both regression lines indicate a positive correlation between g_*sw*_ and Ball Index, representing the fitted data calculated from the leaf-scale measurements, where both of the R^2^ values are higher than 0.90. The slope of the linear regression (g_1_) for AA is about half the slope for FA (Figure 4), and the g_*sw*_ intercept (g_0_) of the linear regression for AA is also smaller than g_0_ for FA (*P* < 0.005).

## 4. Discussion

### 4.1. Aerosol Deposition on Leaf Surfaces

The SEM images of *C. camphora* bring out comparable results with previous research regarding the relation of deposited aerosols and leaf morphology, and the formation of amorphous regions similar to so-called “wax degradation” on the cuticle or close to stomata (Burkhardt, 2010; Burkhardt and Grantz, 2017; Chen et al., 2017). The pattern of hypothetical aerosol layer and amorphous wax degradation have been as well found on the leaf surfaces of *Cryptomeria japonica* (Sase et al., 1998), *Brassica oleracea (Gongylodes Group)* (Burkhardt et al., 2001), *Platanus orientalis L*. (Pourkhabbaz et al., 2010), *Pinus sylvestris L*. (Burkhardt and Pariyar, 2014), *Quercus variabilis* (Mo et al., 2015), and *Vigna radiata (L.) R. Wilczek* (Shabnam et al., 2021). Moreover, research has indicated that identified wax degradation might be actually a mixture of deliquescent aerosols and disturbed wax crystallization; the development of amorphous wax appearance can result from deliquescent salts covering tubular wax fibrils, following the process of (i) the attraction of water vapor by hygroscopicity; (ii) the dissolution of hygroscopic aerosols; and (iii) the resulting mobility and distribution across the leaf surface, leading to the coverage of tubular waxes by amorphous crusts and consequently showing the typical appearance of wax degradation (Burkhardt, 2010; Burkhardt and Pariyar, 2014; Burkhardt et al., 2018). In this study, few larger deposited aerosols on AA leaf surfaces are observed as crystalline, but most of them appear to be amorphous crusts caused by the humidity cycle correlated with the deliquescence of salt and the transpiration of stomata. This phenomenon consists of the previous studies aforementioned.

As for the results of not dissolvable particulate matter and dissolvable aerosol concentration from leaves grown in the greenhouses, it is significant that AA leaves accumulated higher aerosol concentration than FA leaves, regardless of the total aerosol concentration and specific aerosol compounds. Compounds such as Na and Cl may come from sea salts, even though the greenhouses locate a bit distant from the coast (Burkhardt and Eiden, 1990). In general, the dominant aerosol compounds in AA are similar to previous research which was done in the same greenhouse environment (Burkhardt and Pariyar, 2016). Although the epicuticular wax may partially also contain aerosols (Dzierżanowski et al., 2011; Victório et al., 2021), it is neglectable in this study since the focus is on researching the aerosol effects within one species, instead of the quantification and classification of deposited aerosols. The water dissolvable ions contributed about 30% to the overall aerosol mass found on AA leaves, which is in agreement with the reported range of European aerosol composition (Putaud et al., 2010).

**Figure 5:**
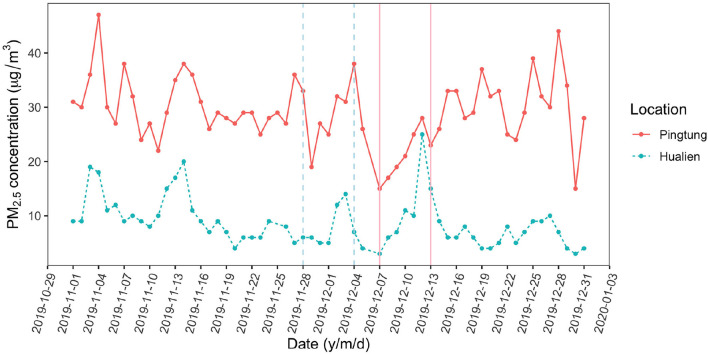
Daily monitoring data of PM_2.5_ in the fields while experiments were conducted (Source: Taiwan Air Quality Monitoring Network, Environmental Protection Administration, Taiwan). The solid line is the Pingtung site (expectedly more polluted) and the dashed line is the Hualien site (expectedly less polluted). The vertical lines indicate the periods while experiments were conducted (Hualien site: 28 November to 4 December 2019; Pingtung site: 7 December to 13 December 2019).

The ionic deposition load on leaves at Pingtung was 1.54 μg/cm^2^, exceeding the amounts on Hualien leaves (1.26 μg/cm^2^) by 22%. This difference was less than expected from long term monitoring data and literature (Lin et al., 2008; Li et al., 2016; Lee et al., 2020). The ionic composition on Hualien leaves was dominated by sea salt (Na, Cl), reflecting the small distance to the sea (50 km in the main wind direction). Nitrate and sulfate are mainly composed of secondary ammonium sulfate and ammonium nitrate from industry (Yang et al., 2017; Shen et al., 2019, 2020). Nitrate, ammonium, and potassium strongly contributed to the composition of particles on Pingtung leaves, whereas the sulfate and magnesium concentrations were higher on Hualien leaves (Figure 2B). The daily monitoring data were extracted from Taiwan Air Quality Monitoring Network, Environmental Protection Administration, Taiwan, in order to inspect the environmental aerosol concentration with an accurate time range (Figure 5). Continuous torrential rain probably caused the strong decrease of PM_2.5_ concentrations shortly before the experiment at the Pingtung site, and also the removal of particles from leaves (Wang et al., 2015), particularly from upper leaf surfaces. However, rainfall itself is also able to contribute to the ion concentrations besides washing off particles; consequently, rainfalls might affect aerosol retention and long term accumulation of ionic aerosols on leaf surfaces, and foliage traits are the more important factors related to these effects (Xu et al., 2017; Pariyar and Noga, 2018; Zhang et al., 2019; Zhou et al., 2020). As an evergreen tree species, *C. camphora* is likely subject to a higher wash off rate of fine aerosols at high rainfall intensities, causing the indistinct aerosol distribution and concentration on leaf surfaces in the Pingtung site (Xu et al., 2019; Zhou et al., 2021). Therefore, this inconsistency is challenging the accuracy of the other field measurements.

### 4.2. Aerosol Impacts on Plant Water Relations in the Field

The high g_*min*_ values and the low abaxial contact angles of leaves at the Hualien site likely are connected effects of aerosol deposition. The g_*min*_ values were more than twice as high and the ratio between adaxial and abaxial contact angles differed compared to the Pingtung site. Although other, e.g., biotic factors cannot be excluded, both effects are likely linked to the relatively high, sea salt dominated deposition at the Hualien site. Normally, NaCl is a kosmotropic salt that does not easily extend on hydrophobic cuticles, so coastal plants are usually not affected too much by sea salt. However, this may considerably change in the presence of detergents, as shown by a strong g_*min*_ increase in a previous experiment, where pine seedlings were sprayed with different salt solutions (Burkhardt and Pariyar, 2014). The detergent reduces the contact angle and promotes stomatal penetration by the salt, i.e., HAS establishment. Several cases of this process in the environment were reported in Italy and Australia, where detergents from close-by landfills caused the coating of sea-spray aerosols leading to the decline of coastal forests (Bussotti et al., 1995). A similar process might actually have played a role at the Hualien site because a landfill in the major source region of NaCl aerosols had been eroded by the sea for several years (Taiwan News, 2018). The occurrence of such detergents on the leaves was not measured and the distance of 50 km is considerable, but still, there is a realistic chance that the high g_*min*_ values and low contact angles were connected with this incidence.

Low contact angles particularly on the lower (abaxial) leaf sides of Hualien leaves point to enhanced deposition of fine, sub-micrometer aerosols, which are less affected by gravity but more by molecular mechanisms. The g_*min*_ parameter describes the uncontrollable water loss of leaves with closed stomata. A g_*min*_ increase is indicative of reduced drought tolerance and reflects the cuticular permeance, but also the contribution of “malfunctioning stomata,” which are linked to aerosols and HAS (Kerstiens, 1996; Burkhardt, 2010). The higher g_*min*_ values indicate that in the case of extended droughts, aerosol deposition might possibly become problematic for the trees at the Hualien site. It is not possible to deduct further impacts of aerosols on the trees from the field measurements. The δ^13^C values between the two field sites cannot be meaningfully compared, as they are influenced by too many different environmental factors, particularly soil water availability, temperature, and VPD.

### 4.3. Aerosol Impacts on Plant Water Relations in the Greenhouse Study

The greenhouse study with equal environmental conditions between AA and FA enables the comparison of single parameters like g_*min*_ or δ^13^C. Differences between the groups can be attributed to the differences between AA and FA aerosol concentrations, as long as the AA and FA plants are physiologically comparable. This requirement was met in the present case, as seen by the comparison of A_*sat*_ and the AC_*i*_ curves, from which the photosynthetic parameters were extracted. These parameters were very similar between AA and FA. The higher daytime respiration R_*d*_ was consistent between AA and FA but was several times higher than at the field sites and in an earlier field study with camphor trees (Kosugi and Matsuo, 2006); possibly due to the effects of the incomparable temperature differences between the greenhouses and the fields, or the inaccuracy caused by different calculators while fitting AC_*i*_ curve data (Sharkey, 2016). It was hypothesized that physiological responses to aerosols would include higher g_*min*_, less negative δ^13^C value, lower leaf water potential at turgor loss (Bartlett et al., 2012a; Maréchaux et al., 2015), and higher proline concentration in the AA compared to the FA greenhouse. With a similar experimental approach, aerosols had caused higher g_*min*_ for *Quercus petraea, Abies alba, Pinus sylvestris* (Burkhardt and Pariyar, 2014; Burkhardt et al., 2018), and *Vicia faba (L.)* (Grantz et al., 2018), as well as less negative δ^13^C for second year *Abies alba* needles, (Burkhardt et al., 2018), while *Helianthus annuus, Pinus sylvestris*, and *Fagus sylvatica* were found to have more negative δ^13^C values (Burkhardt and Pariyar, 2016).

In this study, particularly the results of g_*min*_ and δ^13^C did not confirm the hypothesis. The g_*min*_ results were almost identical between AA and FA greenhouses, which was about half the Pingtung values and only about one-fifth of the Hualien value. A major reason for missing significant differences probably was the small number of repetitions (*n* = 4). This is particularly relevant for the g_*min*_ parameter, where due to high variances and small effects often about 20 repetitions are required to reach significant results. The high variability probably comes from the situation that the water loss by incompletely closed, ‘leaky' stomata is an individual process affecting single stomata, but often is the dominating pathway of water loss in the g_*min*_ measurement compared to water loss across the cuticle (Heinsoo and Koppel, 1998; Burkhardt, 2010; Duursma et al., 2019). A study of *Hedera helix* indicated that 35% of water loss occurred across the incompletely closed stomatal pores and 65% across the other part of the cuticle which is without stomata, and the cuticular transpiration of the stomatous leaf surface was about 11 times higher than the astomatous leaf surface (Šantrůček et al., 2004). Moreover, taking conifer species as research material, it is concluded that the percentage of water loss from stomatal pores of detached leaves might depend on species-specific strategies for conserving water during drought (Brodribb et al., 2014). Because only few studies have found significant correlations between g_*min*_ and environmental factors, other procedures may be more useful under certain conditions (Brodribb et al., 2014; Schuster et al., 2017; Duursma et al., 2019). Under less defined conditions, another possible reason for questioning the reliability of g_*min*_ is the acclimation of plants to the environment. In general, research has shown that plants change the chemical composition of the cuticle while facing water stress, leading to a decreased g_*min*_ value (Bengtson et al., 1978; Premachandra et al., 1992; Macková et al., 2013; Bi et al., 2017). The observation that older leaves have higher g_*min*_ values (Jordan and Brodribb, 2007), might however be caused by the damage of cuticle on old leaves or the increasing contribution of HAS establishment and induced water loss across the stomatal pore (Burkhardt, 2010).

The discrimination value of carbon isotope composition (δ^13^C) provides information on the long term transpiration efficiency of plants, and a lower δ^13^C value is often determined as lower WUE (Farquhar and Richards, 1984; Farquhar et al., 1989; Hubick and Farquhar, 1989; Condon et al., 1992; Cabrera-Bosquet et al., 2007), but requires equal environmental conditions between the compared groups. However, recent studies have focused on more comprehensive and practical conditions instead of an ideal growing environment such as breeding fully fertilized plants in the greenhouse (Conte et al., 2003; Cabrera-Bosquet et al., 2007; Burkhardt, 2010; Berriel et al., 2020; Vogado et al., 2020). Thus, the correlation between δ^13^C and WUE might be influenced by deposited aerosols and HAS, but also by soil water, the nutrient conditions, and the acclimation to stresses (Cabrera-Bosquet et al., 2007; Berriel et al., 2020; Tarin et al., 2020), which is why the field values cannot be compared.

The hypothesis of lower leaf water potential at wilting (i.e., turgor loss point, π_*tlp*_) by aerosols was also not confirmed. π_*tlp*_ is considered another important determinant of ecological and physiological drought tolerance, which is also strongly correlated with the cell solute potential at full hydration (i.e., osmotic potential, π_*o*_) (Bartlett et al., 2012a,b; Banks and Hirons, 2019). Previous research has focused on π_*tlp*_ of plant species such as woody species, crops, and herbaceous grassland species, concluding that this indicator of drought tolerance varied across species and environmental conditions; π_*tlp*_ is as well correlated slightly with several leaf functional traits such as leaf dry matter, leaf vulnerability to hydraulic failure, leaf toughness, and leaf thickness (Maréchaux et al., 2015; Griffin-Nolan et al., 2019). Normally, a more negative π_*tlp*_ increases the functional range of foliar water potential, showing a greater leaf-level drought tolerance (Mart et al., 2016; Banks and Hirons, 2019). Under defined conditions, a more negative π_*tlp*_ would thus mean that the plant had experienced drought stress by aerosols (Navarro et al., 2007; Burkhardt, 2010). This should be further evaluated using experiments with higher numbers of biological repetitions, including the evaluation of an eventual accumulation of proline. Proline is an additional indicator of osmotic adjustment, responding to environmental stress such as water deficit, salinity, heat, and pollutants (Bates et al., 1973; Dolatabadian et al., 2008; Acosta-Motos et al., 2017). In this study, the proline concentration of leaves did not differ with aerosol exposure, and concentrations in both AA and FA were relatively low.

### 4.4. Aerosols and Water Use Efficiency

Aerosols did not decrease WUE, as it originally had been expected. Contrariwise, the VPD curve of the FA plants had higher g_*sw*_ values than AA, which was highly significant. The subsequently calculated Ball-Berry g_1_ parameter for FA was twice the value compared to AA. Because g_*min*_ (which can be considered the g_1_ factor of the Ball-Berry equation; Duursma et al., 2019) was negligible compared to g_*sw*_ for both AA and FA, this means double WUE of AA compared to FA (Equation (4); Miner and Bauerle, 2017). The relationship between g_1_ and WUE is originally linked to intrinsic WUE (A/g_*s*_) but is also indicative of actual (“instantaneous”) WUE (A/E; Franks et al., 2017). According to the original HAS hypothesis (Burkhardt, 2010), AA leaves should have lost more water than FA at the same degree of stomatal opening; and because this additional water loss is not accounted for by CO_2_ uptake, AA leaves should have lower WUE than FA. But probably this is not the full picture and there may be several independent responses to aerosols. In an AA/FA experiment with *Vicia faba (L.)*, aerosol exposure (i.e., AA) had three effects (Grantz et al., 2018):

(i) reduced stomatal apertures of *Vicia faba (L.)* at each level of VPD;
(ii) increased stomatal conductance at comparable levels of aperture;
(iii) lower heterogeneity between apertures of single pores, i.e., reduced patchiness.

In the present study with camphor, the HAS effect of additional water loss at the equal aperture (effect ii) was likely overcompensated by the aperture reduction of AA stomata (effect i). A reduction of stomatal aperture, however, is known to increase the WUE of seed plants, e.g., in response to drought stress (Franks et al., 2015; Guerrieri et al., 2019; Xu et al., 2021; Yang et al., 2021). The measured increase of WUE_*i*_ by aerosols thus indicates a reduction of stomatal aperture, in agreement with the results of the *Vicia faba (L.)* experiment (Grantz et al., 2018, 2020). It is also in agreement with these earlier results that the error bars of the AA data points were smaller than for FA, indicating lower variation, higher coordination between stomatal apertures (effect iii), and less patchiness - a general susceptibility of the *C. camphora* to the stomatal patchiness phenomenon has earlier been reported (Takanashi et al., 2006). The aperture reduction was not directly measured but would have been independently supported if lower δ^13^C values of AA compared to FA leaves were observed. This was not the case, possibly because the results of the VPD curves and the δ^13^C signals were determined by different micro-climatological conditions: The VPD curves were measured within ventilated cuvettes. The δ^13^C values are a time integrated signal of gas exchange, produced under the calm greenhouse conditions with a thick leaf boundary layer surrounding the leaves most of the time; so stomatal responses are decoupled from the environmental VPD and its interaction with deposited aerosols.

Generally, the g_1_ parameter represents a compromise between the costs and benefits of g_*sw*_ relative to the photosynthetic activity of the leaf (Ball et al., 1987; Miner and Bauerle, 2017). The g_0_ is normally defined as either (i) a fit parameter extrapolated as the intercept of the least squares regression between g_*sw*_ and the Ball Index (Ball et al., 1987; Ball, 1988; Collatz et al., 1991), or (ii) the residual conductance when A_*n*_ ≤ 0 (Leuning, 1995). The g_1_ values here were 1.71 (AA) and 3.41 (FA) and, thus, considerably lower than the value of 7.4 observed for *C. camphora* in a field study (Kosugi and Matsuo, 2006). Both g_0_ and g_1_ were at the lower end but still within the range of previously recorded values (Miner et al., 2017; Wolz et al., 2017). Drought affected plants, e.g., *Eucalyptus, Quercus, Zea mays*, and *Helianthus*, often have lower g_1_ and g_0_ values compared with well-watered plants of the same species (Cavender-Bares et al., 2007; Heroult et al., 2013; Zhou et al., 2013; Miner and Bauerle, 2017; Miner et al., 2017). The lower g_1_ value of AA camphor leaves compared to FA can thus possibly be interpreted as aerosol induced drought stress. The reason for the involvement of H_*s*_ for plant transpiration in the original, semi-empirical Ball-Berry model has remained elusive and its relevance was questioned, compared to VPD which seems to be physiologically more meaningful (e.g., Monteith, 1995). The successful H_*s*_ use, however, might well be due to the direct interaction of hygroscopic, deposited aerosols with water vapor on the leaf surface. This kind of interaction is immediate and direct and the method to determine water absorption to specific salts has been used to determine the relative humidity in weather balloons (Wylie, 1955).

## 5. Conclusion

Fine hygroscopic aerosols are ubiquitous. Their presence on leaf surfaces often is not obvious, but the comparison of SEM images from AA and FA greenhouses is a useful method for identifying aerosol related surface structures. Greenhouse and field results behaved differently. The controlled conditions in the greenhouse aerosol exclusion study with camphor seedlings enabled a detailed perspective of aerosol interaction with the stomatal part of the water relations. Aerosols surprisingly caused higher WUE of camphor trees in the greenhouse study, which was the first detailed observation of this kind and may also have relevance on larger scales beyond the leaf-level. The sharply increased WUE of forests over the last century is a globally observed phenomenon and has mainly, but not sufficiently, been explained as a consequence of CO_2_ increase (Keenan et al., 2013; Knauer et al., 2017; Kannenberg et al., 2021). The atmospheric aerosol deposition could be a hidden, contributing factor, which should be investigated.

In the field experiment, the particular challenges came from the cumulative, long-term nature of aerosol effects and the uncontrolled environmental conditions. However, contact angles and g_*min*_ of leaves from the adult camphor trees were probably attributed to the amount and type of aerosols. These parameters seem to be suitable to determine aerosol effects on those parts of plant water relations which are not under stomatal control, i.e., cuticular loss and stomatal leakage by HAS. Marine aerosols, possibly polluted by organic material, might have decreased the drought tolerance of camphor trees at the Hualien site, but additional studies would be needed to confirm this.

## Data Availability Statement

The original contributions presented in the study are included in the article, further inquiries can be directed to the corresponding author/s.

## Author Contributions

S-CC and JB developed the research design. C-JEC, DZ, I-LL, S-CC, and Y-LK contributed to the field work. C-JEC and DZ completed the data processing. C-JEC analyzed the data and wrote the manuscript. All the authors commented on the draft and approved the submitted version.

## Funding

This research was funded by Deutscher Akademischer Austauschdienst (DAAD, German Academic Exchange Service), grant numbers: 57440921 and 57393505. JB was funded by the Deutsche Forschungsgemeinschaft (DFG, German Research Foundation), grant number: 446535617. C-JEC acknowledges support from BIGS - Land and Food.

## Conflict of Interest

The authors declare that the research was conducted in the absence of any commercial or financial relationships that could be construed as a potential conflict of interest.

## Publisher's Note

All claims expressed in this article are solely those of the authors and do not necessarily represent those of their affiliated organizations, or those of the publisher, the editors and the reviewers. Any product that may be evaluated in this article, or claim that may be made by its manufacturer, is not guaranteed or endorsed by the publisher.
